# Effector CD8^+^CD45RO^−^CD27^−^T cells have signalling defects in patients with squamous cell carcinoma of the head and neck

**DOI:** 10.1038/sj.bjc.6600694

**Published:** 2003-01-28

**Authors:** I Kuss, A D Donnenberg, W Gooding, T L Whiteside

**Affiliations:** 1 University of Pittsburgh Cancer Institute, W 1041 Biomedical Science Tower, 200 Lothrop Street, Pittsburgh, PA 15213-2582, USA; 2 Department of Medicine, University of Pittsburgh School of Medicine, Pittsburgh, PA 15213, USA; 3 Department of Pathology, University of Pittsburgh School of Medicine, Pittsburgh, PA 15213, USA; 4 Department of Otolaryngology, University of Pittsburgh School of Medicine, Pittsburgh, PA 15213, USA

**Keywords:** cancer, *ζ* chain, apoptosis, naïve T cells, memory T cells

## Abstract

A subset of circulating T cells (CD8^+^CD45RO^−^CD27^−^) with a naïve phenotype, but mediating effector function, is considered to play an important role in host antitumour defence. To investigate the attributes of these effector T cells in patients with squamous cell carcinoma (SCC) of the head and neck cancer, venous blood was obtained from 39 individuals with cancer and 45 normal controls (NC). Peripheral blood mononuclear cells were isolated, stained with labelled monoclonal antibodies specific for CD8, CD45RO, CD45RA, CD62L, CD27, TCR-*ζ* as well as isotype controls and examined by multicolour flow cytometry. Annexin V binding to CD8^+^ T cells and PMA/ionomycin-induced IFN-*γ* expression were also evaluated in patients and NC. The proportions of CD45RA^+^CD45RO^−^ (naïve) and CD45RA^−^CD45RO^+^ (memory) cells were found to be comparable within the CD8^+^ T-cell subset. However, relative to NC, the frequency of effector CD8^+^CD45RO^−^CD27^−^ cells was strikingly increased in all SCC patients regardless of the disease status (*P*=0.0003). The proportion of these cells was found to increase with age in both patients and NC. In NC, stimulated IFN-*γ* expression was largely restricted to CD8^+^CD45RO^−^CD27^+^ cells, while in patients CD8^+^CD45RO^−^CD27^−^ expressed IFN-*γ* after *ex vivo* stimulation. Expression of the TCR-associated *ζ* chain was decreased or absent in freshly isolated CD8^+^CD45RO^−^CD27^−^ T cells in patients (*P<*0.0001). Annexin V was found to bind to a higher proportion of circulating CD8^+^ T cells in patients than NC (*P<*0.006), and significantly more Annexin V^+^ T cells were present in the effector (*P<*0.0059) than the naïve subset within the CD8^+^CD45RO^−^ compartment. The data indicate that the expanded CD8^+^CD45RO^−^CD27^−^ T cells, which contain precursors of IFN-*γ*-producing T cells, are *ζ*-negative and sensitive to apoptosis in the circulation of patients with HNC.

Recent data from our laboratory and other laboratories indicate that tumour-infiltrating and peripheral T cells of many patients with cancer are primed for apoptosis (Rabinowich *et al*, 1998; Reichert *et al*, 1998b; Lee *et al*, 1999; Reichert *et al*, 2002; Whiteside, 2002), and that the death of effector T cells could be responsible for inadequate antitumour functions. We previously reported that relative to healthy normal controls (NC), patients with cancer have increased proportions of circulating T cells that bind Annexin V (Dworacki *et al*, 2001; Hoffmann *et al*, 2002). This observation suggests that a higher rate of lymphocyte turnover is associated with cancer. Furthermore, we recently observed that CD8^+^ T cells are preferentially primed for apoptosis, suggesting a more rapid turnover of CD3^+^CD8^+^ than CD3^+^CD4^+^ T cells (Hoffmann *et al*, 2002). CD8^+^ T cells are functionally heterogeneous, and several subsets of CD8^+^ T cells are known to contribute to antitumour immune responses. In addition to an effector T cell subset, both naïve and memory CD8^+^ T cells exist within the peripheral CD8^+^ T-cell pool (Hamann *et al*, 1997; Young *et al*, 1997; Sallusto *et al*, 1999; Baares *et al*, 2000).

Human naïve and memory T cells can be identified by the reciprocal expression of the CD45RA or CD45RO isoforms (Young *et al*, 1997). More recent reports indicate that within the CD8^+^ CD45RA^+^(CD45RO^−^) compartment of naïve cells, a subset of effector T cells, which lack the CD27 receptor as well as the lymph node homing receptors CD62L and CCR7, can be identified (Hamann *et al*, 1997; Sallusto *et al*, 1999). These effector-type CD8^+^CD45RO^−^CD27^−^ T cells are thought to be derived from the CD27^+^ precursors in response to antigenic stimulation. They are characterised by a shorter telomeric restriction fragment (TRF) length compared with unprimed cells, express cytolytic activity and abundantly produce IFN-*γ* and TNF-*α* (Baares *et al*, 2000). It has been suggested that this subset of CD8^+^ T cells plays an important role in host antitumour defence.

To further evaluate the role of this effector CD8^+^ T-cell subpopulation in the control of cancer progression, we investigated the frequency and functional characteristics of effector CD8^+^ T cells in a cohort of patients with squamous cell carcinoma (SCC) of the head and neck. We find that, in contrast to normal controls, the frequency of CD8^+^CD45RO^−^CD27^−^ T cells is significantly increased in the circulation of HNC patients. However, these CD8^+^ effector cells have no or low *ζ* expression and are thus unable to signal. They also contain increased proportions of Annexin V-binding cells. The data indicate that the CD8^+^CD45RO^−^CD27^−^ effector T-cell subset appears to be dysfunctional and destined for apoptosis in patients with cancer.

## Materials and Methods

### Patients and controls

Venous blood samples (10–30 ml) were obtained from patients with SCC of the head and neck, who were seen between April 2001 and March 2002 at the Outpatient Otolaryngology Clinic at the University of Pittsburgh Cancer Institute (UPCI). The Institutional Review Board has approved the protocol for collection of patient blood samples. Normal healthy donors (NC) were recruited among the laboratory personnel and other volunteers. A written informed consent was obtained from all individuals participating in this study. Two groups of patients and NC were studied. In total, 28 patients and 38 NC were included in studies of CD8^+^ T-cell subset and analysis of CD3 *ζ*-chain expression. An additional 11 patients and seven NC were included in the evaluation of Annexin V binding to the CD8^+^ effector T cells.

The characteristics of all the patients included in this study are shown in Table 1

**Table 1:** Clinicopathological parameters of the patients with SCC of the head and neck included in the study

		***n***
Age (y)		
Mean (range)	61 (36–82)	
Sex		
Male		33
Female		6
		
Total		39
		
Tumour site		
Larynx		11
Oral cavity		12
Pharynx		10
Hypopharynx		2
Miscellaneous		3
Unknown		1
		
Tumour stage		11
T1		
T2		14
T3		6
T4		5
TX		3
		
Nodal status		21
N0		
N1		7
N2		11
		
Tumour differentiation		align="right"2
Well		
Moderate		29
Poor		7
Undifferentiated		1
		
Prior therapy^a^		25
Surgery		23
Radiotherapy		9
Chemotherapy		3
		
Smoking history		
Yes		32
No		4
Unknown		3
		
Status at day of blood draw		
No evidence of disease		25
Active disease (presurgery)		14
Primary		12
Recurrence		2

a Therapy ended ≥ 2 months prior to the blood draw.

. The cohort of 39 patients included 33 men and six women with a mean age of 61 years (range 36–82). The group of normal volunteers comprised 11 males and 34 females with a mean age of 51 years (24–88). All patients had histologically proven SCC of the head and neck, with 11 tumours originating in the larynx, 12 in the oral cavity, 10 in the pharynx, two in the hypopharynx and three at miscellaneous sites. One tumour was of unknown origin. The histological grades of the tumours were well differentiated (*n*=2), moderately differentiated (*n*=29), poorly differentiated (*n*=7) and undifferentiated (*n*=1). At the time of blood draw, 25 patients showed no evidence of disease, whereas 14 patients were studied either before surgery (*n*=12) or had newly diagnosed lymph node metastases (*n*=2) and were classified as having active disease.

### Cell isolation

Peripheral blood mononuclear cells (PBMC) were isolated by Ficoll–Hypaque density gradient centrifugation, recovered from the gradient interface, washed in Dulbecco's phosphate-buffered saline (D-PBS; Life Technologies, Inc., Grand Island, NY, USA), counted in a trypan blue dye, and either stained for flow cytometry or used for *ex vivo* experiments. In each experiment, PBMC obtained from patients were evaluated together with the cells obtained from at least one normal control.

### Cell activation

PBMC obtained from patients or NC were suspended in AIMV medium containing 10% (v/v) foetal bovine serum (FBS) (Life Technologies, Rockville, MD, USA) at a concentration of 2×10^6^ cellsml^−1^. Cells were stimulated with phorbol myristate acetate (PMA, Sigma Chemical Co., St Louis, MO, USA) at 0.5 ng ml^−1^ and 0.5 *μ*M ionomycin (Sigma) for 4, 12 or 18 h followed by 4 h incubation with 2 *μ*M monensin (Sigma). Cells were then harvested for the determination of intracellular IFN-*γ* expression by flow cytometry. In other experiments, cells were stimulated with anti-CD3 mAb (10 *μ*gml^−1^ Ortho Biotech, Bridgewater, NJ, USA), and incubated for 24 or 48 h in preparation for determinations of *ζ*-chain expression as described below.

### Staining for flow cytometry

Aliquots of PBMC were stained for flow cytometry, using a panel of labelled monoclonal antibodies (mAbs) specific for cell surface-associated lymphocyte antigens as follows: anti-CD8-PE-Cy5, anti-CD45RO-ECD, anti-CD45RA-PE, anti-CD27-PE, anti-TCR*ζ*-PE, anti-CD62L-FITC (all from Beckman Coulter, Miami, FL, USA) and anti-CD27-FITC (Caltag Laboratories, Burlingame, CA, USA). Isotype control Abs IgG1^−^ FITC or IgG1-PE were purchased from Becton Dickinson (San Jose, CA, USA). FITC-conjugated Annexin V was purchased from PharMingen (San Diego, CA, USA) and anti-IFN-*γ*-FITC mAbs from Beckman Coulter (Miami, FL, USA). All antibodies were pretitred on normal PBMC to determine optimal working concentrations. Freshly isolated PBMC were incubated with antibodies for 25 min on ice and washed twice in PBS, containing 4% (v/v) FBS and 0.1% (vv^−1^) NaN_3_. After staining, the cells were fixed with 0.5% paraformaldehyde in PBS prior to flow cytometry analysis. Staining for the TCR *ζ* chain or IFN-*γ* in CD8^+^ T-cell subsets was performed as follows: first, the cells were surface stained, then fixed with 0.5% paraformaldehyde in PBS for 10 min at room temperature and washed once with PBS/FBS/NaN_3_ and once with 0.1% saponin (Sigma, St Louis, MO, USA) in PBS containing 0.1% bovine serum albumin (BSA). The cells were then stained either with anti-TCR-*ζ*-PE, IFN-*γ*-PE or respective isotype control Abs for 30 min on ice in the dark. After the incubation period, the cells were washed once in saponin solution and once in PBS/FBS/NaN_3_ before fixation with 0.5% paraformaldehyde in PBS.

To measure apoptosis, cells were surface stained and then washed once with PBS/FBS/NaN_3_ and once with Annexin-binding buffer (PharMingen), followed by incubation with FITC-conjugated Annexin V for 15 min at room temperature. Flow cytometry analysis was performed within 60 min on a Coulter Epics XL flow cytometer.

### Statistical analysis

Linear regression analysis was conducted to assess the association between the phenotypically defined T-cell subsets and age. Estimated regression parameters were tested for homogeneity between patients and controls. If changes in the subsets were associated with age, differences between patients and controls were adjusted to reflect the imbalance in age between the two groups. If changes in subsets were independent of age, group differences were examined using the Wilcoxon test for two group differences (patients *vs* controls) or the Kruskal–Wallis test for three groups (patients with active disease *vs* patients with no evident disease (NED) *vs* controls). Differences with *P*-values of less than 0.05 were considered to be significant.

## Results

### Distribution of CD8^+^ T-cell subsets in patients and NC

**Figure 1:**
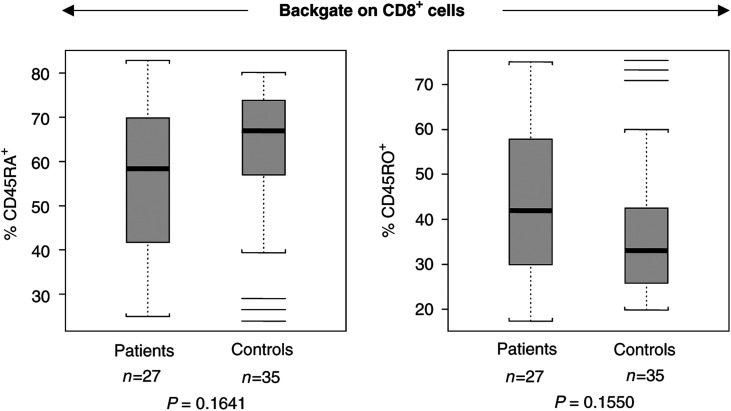
Box and whisker plots showing the percentages of CD45RA^+^ (naïve) and CD45RO^+^ (memory) cells among CD8^+^ T cells in normal controls and patients. The white bar is the median, the black box is the interquartile range (25–75%) and the whiskers extend to 1.5 times the interquartile range.

To study the heterogeneity of the peripheral CD8^+^ T-cell pool, we first evaluated the distribution of memory and naïve CD8^+^ T cells based on the surface expression of the CD45RA (‘naïve’) or CD45RO (‘memory’) isoforms in patients and NC. There was no significant difference in the proportion of CD45RA^+^ naïve CD8^+^ T cells between patients and controls (Figure 1

). In NC, the median (range) for CD8^+^CD45RA^+^cells was 67% (24–80%) compared to 58% (24–83%) in patients. The CD8^+^ CD45RO^+^ memory cells represented 37% (20–75%) of circulating CD8^+^ cells in NC compared to 43% (17–75%) in patients (Figure 1). Although patients generally had fewer naïve CD8^+^T cells and a higher proportion of memory CD8^+^ T cells than NC, these differences were not statistically significant (*P*=0.1641 and 0.1550, respectively; Table 2

**Table 2:** Proportions and mean fluorescence intensity of *ζ* in CD8^+^ T-cell subsets in patients with SCC of the head and neck and normal controls

	**Patients**			**Controls**			
**% Positive cells**	***n***	**Mean**	**s.d.**	***n***	**Mean**	**s.d.**	*****P***-value**
CD8^+^CD45RA^+^(RO^−^)	27	57	±17	35	63	±15	0.1641
CD8^+^CD45RO^+^(RA^−^)	27	43	±17	35	38	±15	0.1550
CD27^−^ in CD8^+^CD45RO^−^	28	48	±19	38	37	±19	0.0003^a^
CD27^−^CD62L^−^ in CD8^+^CD45RO^−^	28	50	±23	38	25	±23	0.0001^a^
CD27^−^CD62L^+^ in CD8^+^CD45RO^−^	28	4	±7	38	4	±4	0.508^a^
*ζ* in CD8^+^CD45RO^−^CD27^−^b	13	194	±256	16	759	±346	<0.0001

a Age-dependent subsets, adjusted means and *P*-values.

b Mean fluorescence intensity.

).

**Figure 2:**
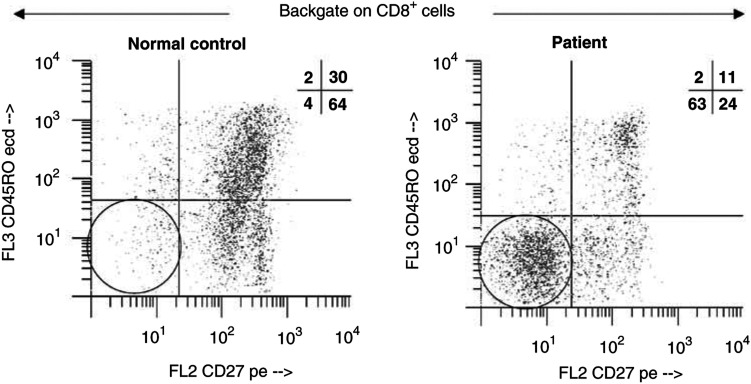
Representative flow cytometry data, illustrating an increase in the proportion of CD27-negative cells among CD8^+^CD45RO^−^ T cells in the patient. The percentages of positive cells in each quadrant are shown in the right upper corner.

We next examined the expression of CD27 molecules on the surface of peripheral blood CD8^+^ T cells. The proportion of CD8^+^CD45RO^−^CD27^−^ cells was found to be significantly greater in patients than in NC (*P*=0.0003; Table 2). The observed enrichment of CD27-negative cells within the CD8^+^CD45RO^−^ T-cell compartment in patients with HNC was of special interest, because the loss of CD27 expression has been reported to identify the effector phenotype (Hamann *et al*, 1997). As illustrated in Figure 2

, CD27-negative T cells within the CD8^+^CD45RO^−^ population were expanded in the peripheral circulation of a patient, whereas considerably fewer of these cells were present in the circulation of an NC.

**Figure 3:**
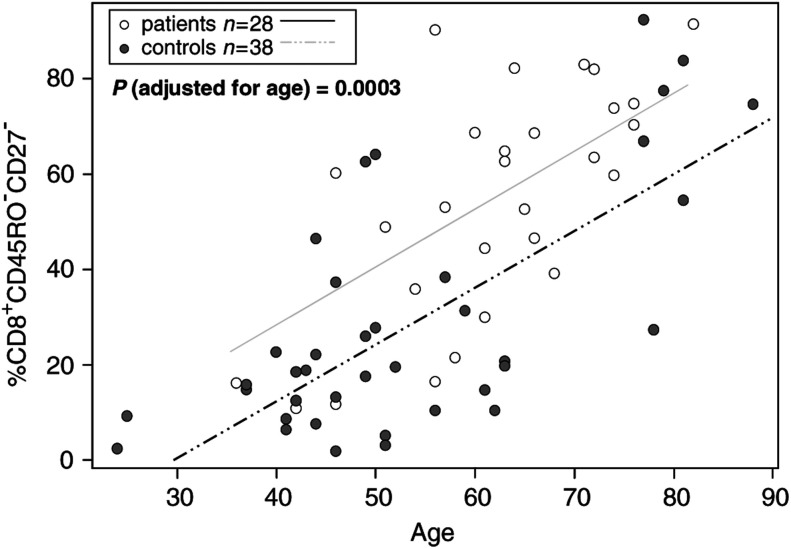
Correlations between age (in years) and the percentages of CD27-negative cells among CD8^+^CD45RO^−^ lymphocytes in the peripheral blood of patients and normal controls. The *P*-value for the significance of differences in the percentage of these lymphocytes between patients and controls has been calculated after adjustment for the age difference between these groups.

The cohorts of patients and NC were not age matched in our study, and it was possible that the observed difference in the proportion of CD8^+^CD45RO^−^CD27^−^ cells between these cohorts was related to age. We therefore compared the patients and NC for changes in this subset of cells relative to age. As Figure 3

shows, the frequency of CD8^+^CD45RO^−^CD27^−^ T cells increased with age in both patients and NC, but the percentage of these cells was significantly higher in patients compared to healthy normal individuals at all ages (*P*=0.0003). We also examined the proportion of the reciprocal subset of naïve CD8^+^CD45RO^−^CD27^+^T cells in the same patients as well as NC, and found that it decreased with age and was significantly lower in patients than in controls (data not shown).

**Figure 4:**
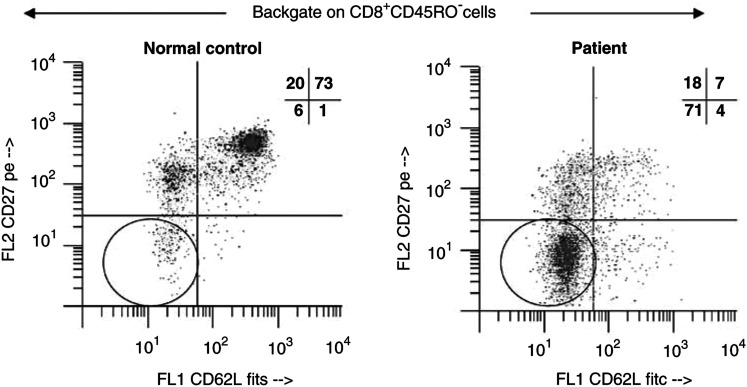
Representative dot plot, showing an increased percentage of CD62L negative expressing cells among the subset of CD8^+^CD45RO^−^CD27^−^ T cells of the patient. The percentages of positive cells in each quadrant are shown in the right upper corner.

In addition to lacking CD27, the effector CD8^+^ T-cell subset was also reported to lack the lymph node homing receptor CD62L (Sallusto *et al*, 1999). In our cohort of NC, the expression of CD62L within the CD8^+^CD45RO^−^CD27^−^ population was found to be very low with a mean±s.d. of 4%±4 ( Table 2). In agreement with the data reported in the literature, the CD27-negative effector CD8^+^ T cells were also CD62L-negative, and the proportion of these cells was highly elevated in the peripheral circulation of patients (50%±23) relative to NC, (25%±23). Representative data for one patient and an age-matched control are shown in Figure 4

.

Within the memory CD8^+^CD45RO^+^ lymphocyte subset, the proportions of CD27-negative and CD27^+^ cells were found to be comparable in patients and NC (*P*=0.38 and 0.39, respectively). The subset of CD8^+^CD45RO^+^CD27^+^ cells decreased with age in both patients and NC, while the reciprocal subset of CD8^+^CD45RO^+^CD27^−^ cells showed an increase with age in both groups (data not shown).

The percentage of effector CD8^+^CD45RO^−^CD27^−^ T cells was not found to be significantly different among patients with active disease or those who had no evidence of disease (NED). Likewise, patients with tumours at different sites or with tumours at distinct stages of differentiation had similar percentages of effector CD8^+^ T cells (data not shown).

### Stimulated IFN-*γ* expression in CD8^+^ T-cell subsets

**Figure 5:**
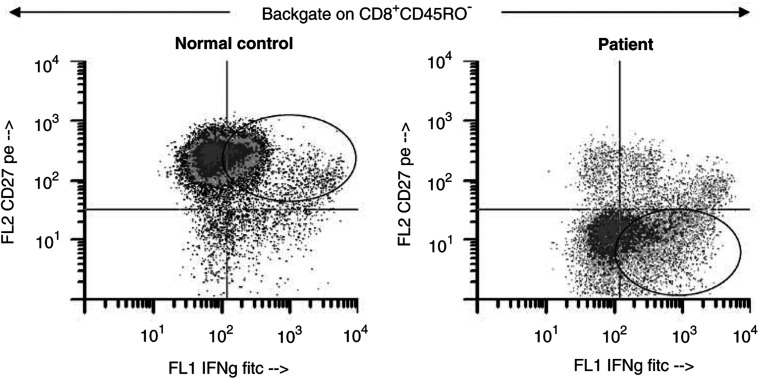
Representative flow cytometry dot plot, demonstrating expression of IFN-*γ* in the effector T cells (CD8^+^CD45RO^−^CD27^−^) of a normal donor and a patient with cancer after 18 h of *in vitro* stimulation with PMA and ionomycin as described in Materials and Methods. Note that effector T cells expressing IFN-*γ* after stimulation are CD27^+^ in NC and CD27-negative in the patient.

PBMC of patients and controls were cultured in the presence of PMA/ionomycin, stained for IFN-*γ* expression and examined by flow cytometry to determine the distribution of precursors of IFN-*γ*-producing cells among CD8^+^ T-cell subsets. Stimulated IFN-*γ* expression was found to be largely confined to CD27^+^ T cells in NC. However, in cancer patients, most of IFN-*γ*^+^ cells were CD27-negative following stimulation with PMA and ionomycin (Figure 5

). While the proportions of IFN-*γ*^+^CD27^−^ T cells observed after *ex vivo* stimulation varied broadly in different patients, the precursors of IFN-*γ*-producing cells were consistently found within the CD8^+^CD45RO^−^CD27^−^ compartment in patients and within the CD8^+^CD45RO^−^CD27^+^ subset in NC. Freshly isolated, noncultured subsets of CD8^+^CD45RO^−^ cells expressed no IFN-*γ* in patients or NC. Thus, the CD27-negative subset, which is expanded in patients, contains precursors of IFN-*γ*-producing effector CD8^+^ T cells.

### Expression of TCR *ζ* in CD8^+^ T-cell subsets

**Figure 6:**
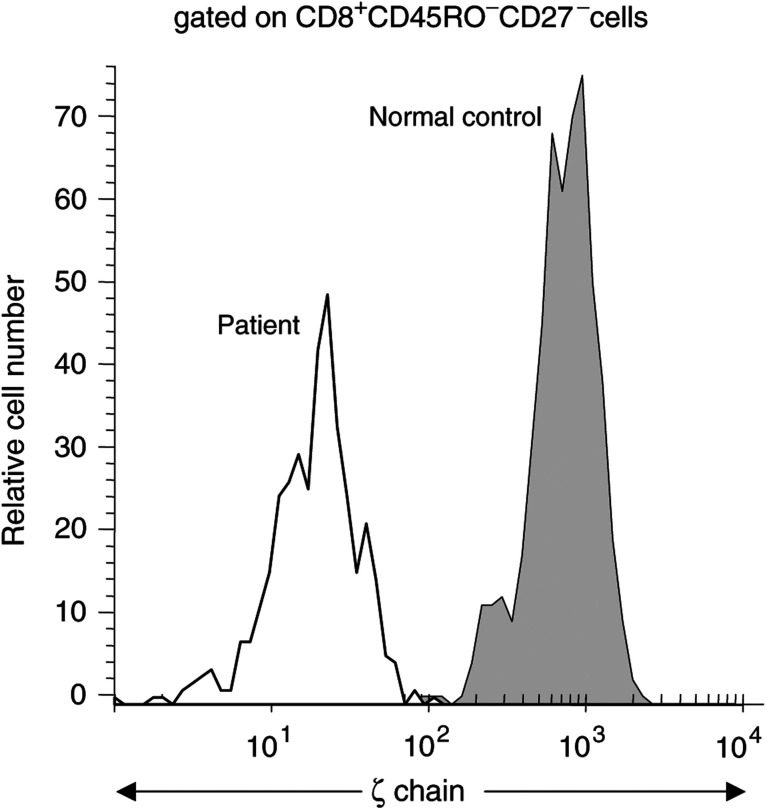
Representative flow cytometry histogram, showing MFI of the *ζ* chain in effector T cells of a patient and an NC. The gate was set on CD8^+^CD45RO^−^CD27^−^ effector T cells. Note the substantial decrease of *ζ* MFI in the patient's T cells.

**Figure 7:**
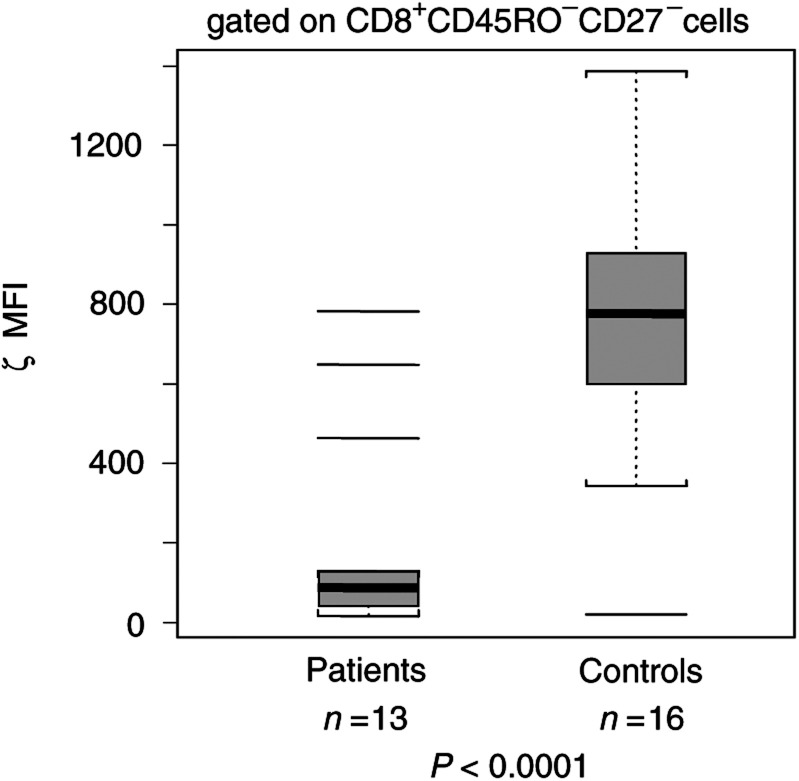
Box and whisker plots showing MFI for TCR-associated *ζ* chain in effector T cells of patients and NC. The gate was set on CD8^+^ CD45RO^−^CD27^−^ effector T cells. See the legend to Figure 1 for an explanation of the box plot.

In order to verify the functional integrity of the TcR signalling pathway in CD8^+^ effector T cells in the peripheral circulation, we next determined the expression of TCR-associated *ζ* chain in CD8^+^CD45RO^−^CD27^−^ effector T cells in an additional 13 patients with SCC of the head and neck and 16 NC. We observed a significantly decreased mean fluorescence intensity (MFI) for *ζ* in patients relative to NC (Figure 6 and Figure 7

), with an MFI of 194 (range 15–782) compared to 760 (range 19–1385) in NC (*P<*0.0001). Thus, although this subset of effector cells was expanded in patients relative to NC, the near absence of *ζ* expression in effector T cells suggested that their signalling via TcR was compromised. Expression of the *ζ* chain was also found to be depressed in the naïve and memory subsets of CD8^+^ T cells in these patients (data not shown). When PBMC of patients and controls were stimulated with anti-CD3 mAb, expression of *ζ* in CD8^+^ T cells was lower in patients than in NC at 24 and 48 h of culture (data not shown).

### Annexin V binding to CD8^+^ effector T cells

**Figure 8:**
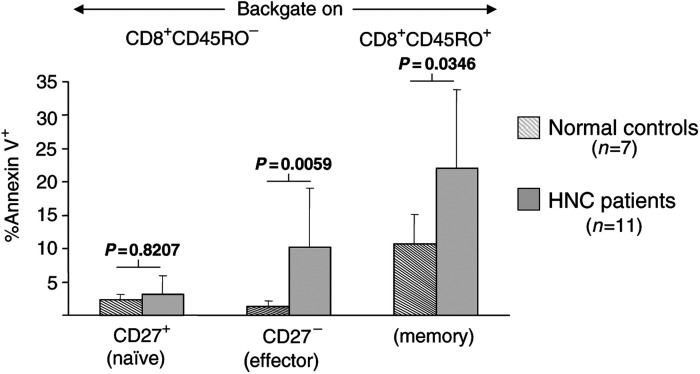
Percentages of Annexin-binding cells among CD8^+^ T cell naïve and memory subsets in patients and controls. Both effector and memory CD8^+^ T-cell subsets in patients with HNC contain significantly more Annexin V^+^ cells than the comparable subsets in controls.

We have previously reported that decreased *ζ*-chain expression could be related to programmed cell death of circulating CD3^+^ T cells in patients with cancer (Gastman *et al*, 1999; Dworacki *et al*, 2001; Hoffmann *et al*, 2002). To investigate whether apoptosis could account for the observed decreased *ζ* expression in CD8^+^CD45RO^−^CD27^−^ effector T cells, we studied Annexin V binding to subpopulations of naïve, effector and memory CD8^+^ T cells in a subset of our patients. In agreement with our previously reported data (Hoffmann *et al*, 2002), we observed that a greater proportion of CD8^+^ T cells bound Annexin V in patients than in NC (*P*=0.006). Furthermore, when Annexin V binding to the cells within the CD8^+^CD45RO^−^ compartment was evaluated in patients, significantly more CD27-negative-Annexin-binding T cells were observed relative to NC (*P*=0.0059), as shown in Figure 8

. Thus, while this subset of effector cells was expanded in patients with cancer, a higher proportion of the cells were in early apoptosis in patients than in NC. There was almost no binding of Annexin detected among the remaining naïve subset of CD8^+^ T cells, while in the memory compartment (CD8^+^CD45RO^+^), the proportion of Annexin-binding cells was increased in patients relative to NC (Figure 8).

## Discussion

Our earlier observations indicated that CD8^+^ lymphocytes are preferentially targeted for apoptosis in patients with advanced cancer (Hoffmann *et al*, 2002). This finding has focused our attention on the subsets of CD8^+^ T cells and their susceptibility to apoptosis. Among the subsets of CD8^+^ T cells, CD8^+^CD45RO^−^CD27^−^ cells are considered to be responsible for effector functions (Hamann *et al*, 1997; Sallusto *et al*, 1999; Baares *et al*, 2000). The phenotypic and functional characteristics of these cells have been extensively investigated and are consistent with their status of antigen-primed effector cells (Baares *et al*, 2000; Hendrics *et al*, 2000). In tumour-bearing hosts, these cells are likely to play an important role in the control of tumour growth. Thus, we were especially interested in the fate of CD8^+^CD45RO^−^CD27^−^ in patients with cancer.

By multicolour flow cytometry, it was possible to differentiate between the naïve, memory and effector subsets of CD8^+^ T lymphocytes, using antibodies to well-recognised surface markers CD45RA and CD45RO in combination with CD27 or CD62L (Hamann *et al*, 1997; Young *et al*, 1997). Strikingly, we observed that the population of CD8^+^CD45RA^+^CD27^−^ T cells was greatly expanded in the circulation of patients with SCC of the head and neck. This finding was readily explainable by the accelerated recruitment of naïve T cells into the effector and memory cell pools. Indeed, the overall proportion of naïve CD8^+^CD45RA^+^ T cells (including effector cells) was somewhat lower and that of memory CD8^+^CD45RO^+^ cells higher in patients than in controls (Figure 1) although these differences did not reach statistical significance. The expansion of the effector cell pool in patients was not found to be an age-dependent process (Figure 3), as it was present in young and old patients. It was viewed by us as a potentially important manifestation of host antitumour defence. Furthermore, *ex vivo* stimulation of patients' PBMC with PMA/ionomycin showed that the expanded CD8^+^CD45RO^−^CD27^−^ subset contained precursors of IFN-*γ*-producing cells, confirming the effector cell status of this population (Hamann *et al*, 1997; Young *et al*, 1997). However, we discovered that practically all of the T cells in the effector subset had low or no *ζ* expression. This finding indicated that signalling via TcR was dysfunctional in expanded CD8^+^CD45RO^−^CD27^−^ cells of patients with cancer. Our results suggest that this subset of effector cells is dysfunctional in cancer patients. While patients' T cells may recover expression of *ζ* in part during *ex vivo* stimulation with anti-CD3 mAb, *ζ* remained low at 24 or 48 h of culture relative to its expression in normal T cells incubated in parallel.

Downregulation of *ζ* expression has been reported in T and NK cells of patients with cancers (Zea *et al*, 1995; Rabinowich *et al*, 1996, 1998; Reichert *et al*, 1998a, 1998b; Kiessling *et al*, 1999; Kuss *et al*, 1999; Whiteside, 1999, 2002; Meidenbauer *et al*, 2002; Reichert *et al*, 2002) and with chronic infections, including HIV (Stefanova *et al*, 1996; Zea *et al*, 1998). A transient loss of *ζ* expression appears to be a normal consequence of signalling via TcR (Valiutti *et al*, 1997). However, in patients with cancer or chronic infections such as leprosy or HIV, *ζ* expression may never recover to equal that in normal T cells (Stefanova *et al*, 1996; Zea *et al*, 1998), and its absence or partial loss in cancer patients appears to be substantially more common in tumour-infiltrating than in circulating T lymphocytes (Otsuji *et al*, 1996). Several different mechanisms have been proposed to explain the loss of *ζ* expression, including production of reactive oxygen metabolites (ROM) by tumour or tumour-associated monocytes (Kono *et al*, 1996; Otsuji *et al*, 1996), or by circulating activated granulocytes (Schmielau and Finn, 2001); apoptosis in the tumour microenvironment (Gastman *et al*, 1999; Hoffmann *et al*, 2002; Whiteside, 2002); production and release by the tumour of *ζ* inhibitory proteins (Taylor *et al*, 2001); the availability of L-arginine in the microenvironment (Taheri *et al*, 2001) or increased degradation of *ζ* in chronically activated T cells (Penna *et al*, 1999). It is interesting to note that the loss of *ζ* was observed not only in the CD8^+^CD45RA^+^CD27^−^ subset of effector cells, but also in other CD8^+^ T-cell subsets of patients with SCC of the head and neck. Therefore, in agreement with our previously reported data, *ζ* downregulation appears to be a generalised phenomenon in cancer patients, which might be mediated by several distinct mechanisms (Kiessling *et al*, 1999; Whiteside, 1999; Dworacki *et al*, 2001).

It is possible that the observed low or absent *ζ* expression in CD8^+^ T cells of patients with cancer is a consequence of normal antigen engagement in this effector cell population, consistent with their antitumour activity. The cells were able to recover responsiveness after *ex vivo* stimulation with PMA/ionomycin, as evidenced by IFN-*γ* expression. However, not all cells responded by IFN-*γ* expression, and in some patients we studied, none responded. It appears that the expanded precursors of IFN-*γ* producing effector cells in patients with SCC of the head and neck are functionally crippled, and that the mechanism responsible for their dysfunction may be low or absent *ζ* expression. In this respect, it is interesting to note that *ζ* is slowly emerging as a biomarker of survival and prognosis as well as responsiveness to immune therapy in patients with cancer (Zea *et al*, 1995; Reichert *et al*, 1998a, 1998b; Meidenbauer *et al*, 2002).

The finding of increased Annexin binding to the CD8^+^CD45RO^−^CD27^−^ cell subset in patients relative to NC indicated that low *ζ* expression might be related to apoptosis, as previously suggested (Gastman *et al*, 1999; Dworacki *et al*, 2001; Hoffmann *et al*, 2002). As indicated above, the subset of CD8^+^CD45RA^+^CD27^−^ effector cells was not the only subset with a higher rate of apoptosis and low *ζ* expression in CD8^+^ T cells of patients with cancer. In these patients, the memory CD8^+^ compartment was similarly affected. Only the naïve CD8^+^CD45RO^−^CD27^+^ subset of T cells showed a relatively low rate of apoptosis, which was comparable to that of normal naïve cells. Although the CD8^+^CD45RA^+^CD27^−^ effector cell subset was not selectively targeted for apoptosis, *ζ* downregulation, greater sensitivity to apoptosis and the concomitant decreased functional potential of the effector cells, which are normally responsible for antitumour activity, is likely to have a negative impact on the host response to the tumour.

The seemingly contradictory observations of the expanded effector cell pool and increased apoptosis as well as *ζ* degradation within this CD8^+^ subset could be reconciled by considering lymphocyte turnover in a tumour-bearing host. Under conditions of chronic antigenic stimulation, lymphocyte turnover is likely to be increased owing to an increased rate of antigen-driven expansion and activation-induced cell death (AICD), as previously suggested (Van Parijs and Abbas, 1998). Hence, the expansion of the effector cell subset in patients with cancer could be viewed as a natural consequence of response to tumour-associated antigens. The concomitant loss of function and death of activated T lymphocytes are compensated for by the sequestration and release of new T cells from the bone marrow stores (Tough and Sprent, 1994; Mackall *et al*, 1997). However, the pool of naïve CD8^+^ T cells is not increased because of their rapid maturation and transfer to the antigen-experienced compartment. This series of events is consistent with a high rate of lymphocyte turnover in patients with cancer, not unlike that seen in patients with HIV (Mohri *et al*, 1998; Hellerstein *et al*, 1999). The obvious downside of such rapid turnover in T cells is that the normal processes of maturation and differentiation of effector cells are disturbed, possibly leading to ineffective immune responses. Further studies will be necessary to confirm directly the hypothesis linking signalling defects we observed with apoptosis and rapid lymphocyte turnover in patients with cancer.
